# TensorMoG: A Tensor-Driven Gaussian Mixture Model with Dynamic Scene Adaptation for Background Modelling

**DOI:** 10.3390/s20236973

**Published:** 2020-12-06

**Authors:** Synh Viet-Uyen Ha, Nhat Minh Chung, Hung Ngoc Phan, Cuong Tien Nguyen

**Affiliations:** 1 School of Computer Science and Engineering, International University, Ho Chi Minh City 700000, Vietnam; ITITIU17012@student.hcmiu.edu.vn (N.M.C.); hungpn17@mp.hcmiu.edu.vn (H.N.P.); ITITIU18172@student.hcmiu.edu.vn (C.T.N.); 2 Vietnam National University, Ho Chi Minh City 700000, Vietnam

**Keywords:** computer vision, background modeling, foreground extraction, Gaussian Mixture Model

## Abstract

Decades of ongoing research have shown that background modelling is a very powerful technique, which is used in intelligent surveillance systems, in order to extract features of interest, known as foregrounds. In order to work with the dynamic nature of different scenes, many techniques of background modelling adopted the unsupervised approach of Gaussian Mixture Model with an iterative paradigm. Although the technique has had much success, a problem occurs in cases of sudden scene changes with high variation (e.g., illumination changes, camera jittering) that the model unknowingly and unnecessarily takes into account those effects and distorts the results. Therefore, this paper proposes an unsupervised, parallelized, and tensor-based approach that algorithmically works with entropy estimations. These entropy estimations are used in order to assess the uncertainty level of a constructed background, which predicts both the present and future variations from the inputs, thereby opting to use either the incoming frames to update the background or simply discard them. Our experiments suggest that this method is highly integrable into a surveillance system that consists of other functions and can be competitive with state-of-the-art methods in terms of processing speed.

## 1. Introduction

With static cameras, existing literature in computer vision has accounted for a wide range of applications that include vision-based traffic surveillance [1,2], pedestrian tracking [3,4], behavior analysis [5], and many more that have been nothing short of magnificence. Among many applications, background modelling has emerged as a critical task due to its capability in extracting information of interest. Particularly, as a background model aims to segregate static views known as “backgrounds” that are without any objects in motion (e.g., motorbikes, cars, pedestrians, etc.) from inputs, it is used to facilitate extractions of motion information of interest (called “foregrounds”) when they appear. When given a sequence of images (e.g., a video), the formulation of excellent backgrounds requires, at minimum, three conditions: the stability of the recording camera, the illumination from either direct or indirect sources in essential amounts, and the consistency of that image sequence. However, in real-life scenarios, the adequacy of those requirements is not always available due to various forms of noises and even errors (e.g., camera recording malfunctions). Thus, an especially relevant issue in the research into background modelling is the detection of changes for addressing variations among scenes. By highlighting informative regions, it is possible to construct the backgrounds and filter out non-essential areas that would only befuddle modelling. Essentially, background images are susceptible to several particular situations:

– *Illumination transformations*: they are ambient and gradual variations in terms of intensity commonly experienced in various outdoor scenes. They challenge background models to adapt accordingly; otherwise, foreground extractions will become ruined by erroneous pixels [6].
– *Dynamic contexts*: they are slight and continuous fluctuation of background scenes (e.g., water surface rippling, waving tree, etc.). Because backgrounds are static, they can produce a massive amount of false detection in foregrounds.
– *Bootstrapping*: this occurs when a period of clean background image without moving objects is hardly available during model training, as there are always some moving objects in the scene, such as people or vehicles. Unless the background model can learn to use the given data and extrapolate into a suitable background, it will fail.
– *Camouflage and slow-moving foreground candidates*: camouflaging effects are cases where foreground regions are absorbed into background due to some similarity between moving objects and background scenes. When foreground objects are slow-moving or become motionless for too long and be perceived as part of the background, obviously, they gradually riddle background predictions with non-essential components. These are challenging scenarios for distinguishing foregrounds from their background.

Recent research has introduced an extensive variety of Gaussian Mixture Models (GMM) on background modelling at pixel-level processing [7,8]. These schemes aim to represent background images by employing the probability density function, known as the Mixture of Gaussians. Specifically, at every pixel location, this function is used in order to construct the temporal statistics of color intensities for the extraction of a background color intensity. This multimodality allows for rapid adaptation to contextual dynamics and it makes GMM popular for an assortment of deployed applications. Meanwhile, there are deep neural networks (DNN) that have proven their effective generalization abilities on their use of distributed representations and processing in hidden layers for foreground detection [9,10]. However, there is an issue with the lack of transparency in their topological structure and computational expenses that makes them less pragmatic, despite their performances in accuracy. It entails that, under continuously changing scenarios in terms of context where unsupervised approaches generalistically perform without pretraining, deep learning methods essentially require the models to be trained for all perspectives in order to reliably segment object motions. Because DNNs’ architectural parameters approximate the neural mapping functions in the non-transparent fashion via gradient approaches, their training is difficult and can fall into local extrema, especially with the lack of data, whereby the model may not be able to generalize for all pre-arranged scenarios, as indicated in the experiments of DeepBS [11] via only 5% of CDnet-2014 data. Another example is illustrated in Cascade-CNN [12], where the authors attempted to only generalize to frames of the same video (and not to other videos) via overfitting a limited number of samples that are used for training. Furthermore, tuning and modifications on DNNs are known to often have to go through repeated heavy training with possibly more data in order to reach the appropriate degrees of generalization for the model to adapt to constantly changing views, which was indicated by Angelov et al. [13]. In general, according to Bouwmans’s work [14], in utilizing the high accuracy and resource utility of the computationally expensive DNN approaches in the general theme, there are still several challenges to empirical studies into background modelling with the DNNs, which stem from the following critical reasons:

– *The absence of a realistically large-scale dataset:* this is marked by the short supply of a balanced coverage of all challenges. DNNs emerge as reliable decision-makers by modelling a substantial number of weak statistical regularities in the relationship between observed spectacles and their corresponding target foreground masks. Therefore, without proper considerations of all cases, it entails that the true properties of scenes are not sufficiently acquired from the sampling peculiarities of the training set, even though the incurred computational expenses with DNNs are very high.
– *The lack of common models for different contexts:* in cases of scene changes with high variations, incorrect updates pervert the probabilistic distribution of the background model and generate erroneous foreground images. Accordingly, universal models appear as an overwhelming necessity, because they are architected to tackle complex circumstances holistically. A good background model must sustain the balance between precision and accuracy in prior observations and segmentation consistency, whilst ignoring inessential variation in observed scenes in order to avoid model corruption [15].

In order to alleviate those trade-offs, the purpose of this article is to propose a novel method: a context-driven background modelling approach that utilizes a variant of GMM, with an adaptive learning thresholding scheme along a dual-state process of maintenance policy. The key idea is that our unsupervised model is implemented in a tensor-based framework in order to resolve the tension between interpretability, computational complexity in DNNs, and generalized adaptation in universal background models. The use of this scheme makes multivariate statistics of observed scenes parallelizable on GPU in spatial perspective with highly efficient tensor-driven calculations. Furthermore, in order to diminish fallacious updates of the model regarding dynamic scene variation, we regulate the uncertainty degree of the estimated background and selectively update on statistical parameters, making our model both robust and generalizable. Our contributions are encapsulated, as follows:

– First, instead of unravelling how a DNN makes decisions, we developed a single-pipelined, tensor-based, explicable computational graph TensorMoG, which explicitly presents the mathematical foundation of GMM in background modelling. The crux of TensorMoG is that it parallelizes the pixel-wise statistical learning in background distributions while only using tensors, like a DNN, as it maneuvers recurrently.
– Second, we incorporates an adaptive learning for scene variation threshold using entropy estimation to evaluate the stability of the background model. This acts as the signaller when the model enters a period when background prediction is influenced enormously, such as sudden illumination changes.
– Third, we proposed a mechanism of dynamic scene adaptation with the suspension of primary progressions and the utilization of implicit updates in order to minimize the presence of distortions among essential background components. These implicit updates serve to neglect predictably unnecessary variations lest they ruin predictions, or otherwise adopt them as necessary components in the background model when their dynamics are adequately stable.

The rest of this article is organized, as follows: Section 2 presents an overview of the related work in the field. This will be followed by Section 3, which introduces our contributions in order to deal with the deviations in scenes. Next, we discuss the qualitative results and benchmark numerical results in order to corroborate the advantages of our method with other state-of-the-art methods while using currently available datasets in Section 4. Finally, conclusions are drawn in Section 5.

## 2. Materials and Methods

Background modelling has experienced many advancements over the years of research. The arrays of methods are so extensive that, despite various attempts at categorizing them [16,17,18], an all-encompassing taxonomy that is widely recognized by the research community is still very difficult to achieve [6]. The deployment of background modelling in real-life scenarios obviously requires robustness, extensive adaptability, good resource utilization, and fast processing. From published literature of various approaches, there are two categories that prominently meet these criteria of research into making applications: statistic-based and neural-network-based. Statistics-based methods exploit the statistical properties of visual features with an assumption that the chronicle of intensity values of a pixel can be characterized by probabilistic models. These approaches maintain high adaptation and are robust to dynamic background with varying motions through simple algorithmic processing. Neural-network-based methods formulate the underlying patterns of backgrounds and the effect of scene dynamics by performing a data-driven learning on a topology of neural networks. These techniques exploit non-linear statistical data modelling on parallel distributed computing paradigms, utilizing these modern multi-processing technologies in order to effectively handle scene-specific segmentation in video sequences of complex motions. Neural-network-based methods are very promising due to the focus into deep learning from the research community thus far.

A variety of background subtraction methods have been introduced to tackle challenges that are mentioned in Section 1. Among the statistics-based models, the model of Mixture of Gaussians has been widely employed so far. Stauffer and Grimson [19] proposed a pioneering research that used multiple, adaptive Gaussians that were accompanied by K-means approximation to model dynamic backgrounds. This work is followed by many improvements of the GMM that were published to be more adaptive to critical situations. Lee [20] introduced an incremental EM algorithm via a recursive filter that enhances model convergence for dynamic Gaussian distributions with temporal adaptability at every frame. A variant of GMM with Dirichlet process [21] was presented by Haines and Xiang with Student’s t-distribution in order to infer per-pixel update. The authors used the learning scheme of continous update to degrade old information and model a new background when visual scenes evolve significantly. A Time-Adaptive, Per-Pixel Mixtures Of Gaussians (TAPPMOGs) [22] was extended with a general framework of corrective feedback to rapidly adapt to sudden uninteresting scene changes and better reflect longer-term, persistent features of scenes. Another adaptive GMM [23] inspects the efficiency of CIE L*a*b* color space with dynamic learning rate in the update of model parameters. The experimental results displayed an increase in the performance of the existing MoG algorithm while presenting a trade-off between speed and accuracy. However, the downside of the approach lies in the sequential estimations of background models, which not only penalizes running time, but also highlights an under-utilization of cutting-edge parallel technologies. Using single GMM, a novel work conducted by Zhou et al. presented a Hypothesis on Degradation Modification based on Co-occurrence Pixel-Block Pairs, CPB-HoD [24], to resist background changes for foreground segmentation under dynamic scenes. The experimental records showed that their method yields fairly good results under challenging environments, such as illumination changes and background motion, when the update of background model follows the designed strategy of pixel-correlation. Nevertheless, the authors have not yet examined the effectiveness of the approach with an on-line mode. Later studies took spatio-temporal analysis into consideration for background modelling. Lu et al. [25] proposed a model that contructs background with a block-based GMM in order to address the “ghosting” problem in scenes. A novel flux-tensor-based scheme, FTSG, was proposed by Wang et al. [26] in order to exploit spatio-temporal tensor formulation and escalate high adaptation to dynamic scenes. The strategy aims to decrease false positives and false negatives from the resulting moving foreground mask via fusing an extended flux tensor moving object segmentation algorithm, split background/foreground Gaussian models, and multi-source appearance similarity comparison. The approach improved the accuracy of the moving object discrimination task. However, the authors have not yet explored the parallelized version of this tensor-based algorithm. On a side note, motivated by compressive imaging, Cao et al. proposed a novel tensor-based robust principle component analysis for background subtraction, TenRPCA [27]. Under a tensor framework, the approach decomposes a video volume into backgrounds while using spatial-temporal correlations and foregrounds via spatio-temporal continuity. An investigation on synthetic and real-world data showed that the approach demonstrates the superiority over the existing state-of-the-art methods in background/foreground reconstruction, but the authors found that it is a practical challenge when an online version of the model for the complex background (e.g., illumination change, unpredictable weather, and the motion of cameras) has not yet been facilitated sufficiently. As a tensor-driven approach, the published work has shown promising accuracy results, whilst the parallelization of the optimization algorithm is still being investigated in order to address the computational burdens. Chen et al. [28] developed a spatio-temporal smoothing technique in terms of Gaussian kernel density transform to reduce the sensitivity of the model to fast illumination changes. The use of spatial relation of the Wronskian in GMM [29] mitigated misclassifications of foreground pixels and demonstrated robustness to background variations. In addition, a GMM model with a superpixel hierarchy proved spatial and temporal consistency in lighting changes and cluttered scenes [8]. Overall, models of the statistical approach were developed with explicit probabilistic hypotheses. This manner eliminates high-frequency variation, and it maintains a temporal analysis of background models regarding gradual deviation over the time. However, using these heuristic adaptations imposed high computation with complex operations. The evaluation of scene changes is also chosen by a compromise between high adaptability and too rapid convergence to dynamic variation of illumination. In practice, intervention in update process of the model is a sensitive concern. Even though previous work has addressed this issue, the performance is a crucial requirement for any background models in practical systems.

Recent years have already seen many sophisticated methods of learning while using the neural networks [30] in this research field. Deep learning models has a remarkable ability in background generation to cover various dynamics of outdoor environments with a series of layers. A context-encoder [31] was proven to be feasible for modelling the background of a motion-based video by learning visual features of scene context to construct the overall scene of a video. Xu et al. [32] used an adaptive Restricted Boltzmann Machine, which performs approximate learning with an aim of capturing the temporal correlation between adjacent video frames to construct background. Tao et al. [33] showed an augmented version of a generative architecture BM-Unet with unsupervised training to produce background image via a probabilistic heat map of the colour values. The power of the deep learning models also gave attention to background subtraction and foreground detection enhancement. Perceiving the gap of temporal perception in conventional methods and the spatial perspective in modern semantic techniques, Liang et al. exploited the attention mechanism in a spatial-temporal model, called STAM [34]. The method benefits from the combination of statistical features and high-level spatial features that were learned by deep neural networks, which improve the accuracy via taking the rich temporal features of motions in successive frames into account. The approach is theoretically interesting, but the large computational resources it requires makes it costly to deploy in real world. Published work from Babaee et al. [11] was presented in order to make the deep learning methods more universal, i.e., more generalized to various scenarios. The authors enabled a deep Convolutional Neural Network (CNN), DeepBS, in order to exploit spatial features from image-background pairs from various video sequences, then performed a consistent segmentation via a spatial-median classifier. In this work, the measure of background dynamics decreased the sensitivity of the model regarding unseen video sequences of dynamic background, suggesting promising performance, but the method’s adaptability is reliant on construction of contextual backgrounds with additional computational DNN complexity. Nguyen et al. [10] introduced a framework that combines the sample-based background model and a feature extractor that was trained by a triplet network, which was capable of producing high-level representations, in order to produce motion masks. A major advantage of the framework pertains to its good performance on a variety of outdoor and indoor scenes and even on some unseen real-world data. Similarly, with the purpose of handling various dynamic scenarios of observed scenes, Lim et al. proved the effectiveness of deep-learning approach with a encoder–decoder scheme under a triplet framework in the encoder part, FgSegNet [9], in order to embed an image in multiple scales into the feature space and use a transposed convolutional network in the decoder part to learn a mapping from feature space to image space. The method is robust in challenging situations, including illumination changes, background or camera motion, camouflage effect, and shadow. It can be deployed in both indoor or outdoor scenes. FgSegNet achieves highly accurate results on overall evaluation of the CDNet 2014 dataset. Dealing with the shortage of foreground training examples, the authors performed an extensive research with a new version, FgSegNet_v2 [35]. The later variant alleviates the requirements of ground-truths labeling burden via proposing feature fusion inside FgSegNet’s feature pooling module and gives state-of-the-art performance in motion analysis. However, its compromise between accuracy and computation power makes it highly challenging for deployment in real-world scenarios, as it would require state-of-the-art hardware configurations. Wang et al. [12] contributed an end-to-end, multi-resolution CNN, coined Cascade-CNN, which models learning the presence of the background and discriminates regions of moving objects in a semi-automatic paradigm. Experimented on the large scale dataset of video sequences, the method gives high accuracy, but the accuracy comes from overfitting, as the method is re-trained for each video with only a small set of frames. This is due to the fact that the goal of cascade CNN is to accurately automatically label the moving object regions and reduce manual labeling. Zeng et al. [36] proposed an augmentation to a CNN, such as the VGG-16, in order to extract and combine deep and multi-scaled features from its layers for the segmentation of object motion. Thus, instead of using separate models for modelling and updating the background, both processes can be simplified by one network.

Overall, although neural-network-based methods have proven to be successful in background subtraction with scene-specific dynamics, most of them reveal limitations of supervised learning, which refers to a large amount of labelled background for video sequences. These deep visual models were proposed to cope with translations, so they are typically trained on a variety of empirical scenarios in order to generalize across different kinds of scene variations. For practical purpose, they can only reliably process certain types of trained video views, and need to be retrained for unseen scenarios [18]. In addition, the amount of time and memory consumption becomes tremendous in real applications, showing neural-network-based approaches as only an interesting theoretical point for the less resourceful. On the other hand, while traditional statistics-based techniques adopted straightforward literature with a fair generalization of contextual sensitivity well, they are mostly designed in sequential paradigms and are less relevant to modern multi-processing technologies. Hence, one reasonable solution to this dilemma is our development of a tensor-based model that explicitly formulates data-driven statistical analysis in order to generalize viewpoints effortlessly, whilst covering comparative computation cost via a high-dimensional input-output model computed with GPUs.

## 3. Proposed Method

### 3.1. Outline of the Proposed Method

**Figure 1:**
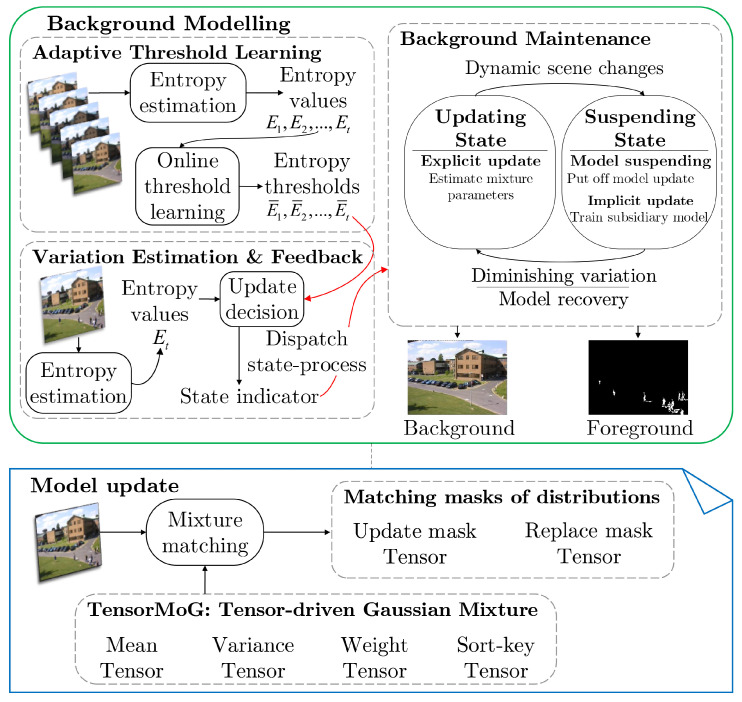
The workflow of our proposed method.

In modeling backgrounds, with an input frame $I_{t}$ at time step *t*, we algorithmically process it via the single-pipelined, tensor-based, explicable computational graph TensorMoG, which explicitly presents the mathematical foundation of GMM in background modelling. From the re-derivations of the foundational computations of GMMs for background modeling in the technologically parallelizable form, the crux of TensorMoG is such that it can perform statistical learning in background distributions simultaneously for multiple pixels. TensorMOG uses four tensor-driven variables, as seen from the bottom picture in Figure 1: Mean, Variance, Weight, and Sort-key tensors for its processing. Whilst the Mean, Variance, and Weight tensors are responsible for constructing pixel-wise Mixture of Gaussians, the Sort-key tensor is used to indicate the degrees of relevance and importance of Gaussian components within each mixture. Supposedly given an imaging source of resolution H×W, where *H* and *W* are the height and width, respectively, let *N* be the number of Gaussian components for each mixture, the dimension of the Variance, Weight, and Sort-key tensors are N×H×W×1, whereas the dimension of the Mean tensor is N×H×W×3, where number 3 accounts for the RGB color space. These tensors are utilized in the mixture matching scheme of the tensor-driven algorithm, where two tensor masks are constructed in order to selectively update the tensor variables (i.e., the Update Mask Tensor, which is responsible for updating the variables with relevant information, and the Replace Mask Tensor, which is used for replacing the most obsolete values with new information).

Next, at every time step *t*, we calculate an entropy estimation $E_{t}$ to evaluate the stability of the current background model, as shown in the left portion of the top picture in Figure 1. Here, we incorporate an online adaptive learning strategy for scene variation threshold ${\bar{E}}_{t}$ while using the value $E_{t}$ along with past entropy estimations. This acts as the signaller when the model enters a period when background prediction is influenced enormously, such as sudden illumination changes, where the values $E_{t}$ and ${\bar{E}}_{t}$ are compared and a decision is drawn.

The global state of the model one of two predefined states, depending on the state indicator from comparisons between $E_{t}$ and ${\bar{E}}_{t}$, as illustrated by the right side of the top picture in Figure 1:

- *Updating State*: the primary stable state where TensorMOG explicitly estimates the tensor mixtures’ parameters at each time step. The absolute difference between $E_{t}$ and ${\bar{E}}_{t}$ is tolerably small for this state to be maintained, or dispatched from the suspending state via model recovery as the context is undergoing diminishing variations.
- *Suspending State*: the auxiliary updating state where the main background modeling progressions of the background tensor mixtures’ parameters are suspended, but a subsidiary model is enabled in order to adaptively learn and replace the original. The absolute difference between $E_{t}$ and ${\bar{E}}_{t}$ is durationally and intolerably large for this state to be dispatched, the scene is expected to be undergoing dynamic scene changes in this case.

This is the mechanism of dynamic scene adaptation with the suspension of primary progressions and the utilization of implicit updates in order to minimize the presence of distortions among essential background components. These implicit updates serve to neglect predictably unnecessary variations lest they ruin predictions, or otherwise adopt them as necessary components in the background model when their dynamics are adequately stable. We shall discuss in greater details the computational graph of TensorMoG in Section 3.2, the online adaptive learning strategy for scene variation threshold in Section 3.3, and the dual-state transitioning processes in Section 3.4.

### 3.2. Gaussian Mixture Model with Tensor-Driven Framework

According to Zivkovic [37], given a learning data set $\chi_{T}=\left\{x_{1},x_{2},\ldots ,x_{T}\right\}$ over a period *T*, each pixel at time *t* is modelled by a probabilistic model mixture of *N* Gaussian distributions:

(1) $$\begin{array}{cc} P\left(x_{t}\right) & =\sum_{n=1}^{N}P\left(G_{n,t}\right)\cdot P\left(x_{t}|G_{n,t}\right)\\ \; & =\sum_{n=1}^{N}\omega_{n,t}\cdot \eta_{n}\left(x_{t};\mu_{n,t},\sigma_{n,t}^{2}I\right) \end{array}$$

where the *n*th Gaussian component in the mixture is estimated with a prior probability $P(G_{n,t})$, also denoted as $\omega_{n,t}$, in order to indicate the proportion of data accounted for by that component. Moreover, each component in the mixture is defined with a probability density function $P(x_{t}|G_{n,t})$ formulated as a normal distribution $\eta (x_{t};\mu_{n,t},\sigma_{n,t}^{2}I)$, where $\mu_{n,t}$ and $\sigma_{n,t}^{2}$, respectively, denote the estimates of the mean and variance describing the *n*th Gaussian component. For statistical learning, soft-partition, where the single best-match component is selected for parameter update, is employed to avoid multiple mixture Gaussian components being considered for a single sample. Subsequently, the model coefficients ($\omega_{n,t}$, $\mu_{n,t}$ and $\sigma_{n,t}^{2}$), as represented by $\theta_{n,t}$, of the chosen distribution are adjusted via an approximation of Expectation Maximization. Given that ${\bar{\theta }}_{n,t}$ is an observed/assumed value for update, the adaptation rate is controlled with a global coefficient α, as follows:

(2) $$\theta_{n,t}=\alpha \cdot {\bar{\theta }}_{n,t}+\left(1-\alpha \right)\cdot \theta_{n,t-1}$$

In our work, we build a tensor-driven variant of MoG following this strategy, which is called TensorMoG. We employ an incremental EM approximation with recursive update into a high-dimensional lattice, where we present spatial arrangement of model parameters in forms of tensor blocks to capture the parallelism of pixel-level learning, in order to exploit the fast temporal adaptability in mixture learning. Accordingly, the core of the framework is a two-fold process: mixture component matching and statistical learning. Much like its traditional counterparts, the input to our tensor-driven architect is a fixed-size RGB image. We use high dimensional variables of variance $\sigma^{2}$ and weight ω that are with the same dimension of N×H×W because our proposed model is also transparently explicable for its core of statistics. Similarly, the dimension of the mean tensor μ in the model is defined to be compatible with the color space of $\chi_{T}$.

With each input image $I_{t}$, we define a matching convention that is based on Euclidean distance in the RGB color space to measure three-channelled consistency where observed intensity falls within a ratio τ of standard deviations of mixture components at each pixel. Rather than performing iterative pixel-wise analysis, it is equivalent to augment the investigation of variables with spatial resolution while using the squared broadcasting subtraction between $I_{t}$ and the mean tensor at the same time step $\mu_{t}$:

(3) $$M_{t}=\phi \left(\tau \cdot \sigma_{t}^{2}-\Delta_{t}\right)$$

where

(4) $$\Delta_{t}={\left(I_{t}-\mu_{t}\right)}^{2}⊗\kappa$$

A model of *N* Gaussian components is required at each pixel within $I_{t}$ in order to examine intensity distance; so, we need to broadcast input frame to fit into the distribution-plane of the mean tensor. Equation (4) denotes that element-wise estimation is performed in the domain of tensor maps, while using convolution with κ, an all-ones vectorized kernel. We apply a threshold-based activation function $\phi \left(\cdot \right)$ in order to produce matching mask denoted $M_{t}$, which indicates distributions matching its corresponding values. Using ${\hat{M}}_{t}$, which is summarized from $M_{t}$, denotes whether or not a pixel location can potentially have its current MoG state matched. In order to have a good grasp of the importance of a component in the mixture, we use a different treatment of weight updates with a ratio of $\omega_{n,t}/\sigma_{n,t}$ in terms of a sort-key tensor ψ. This is the manner of weighting components within a mixture at each pixel by valuing high-weighted, low-spread distributions in the mixture, thereby spotlighting the most significant distribution that contributes to the construction of backgrounds.

**Figure 2:**
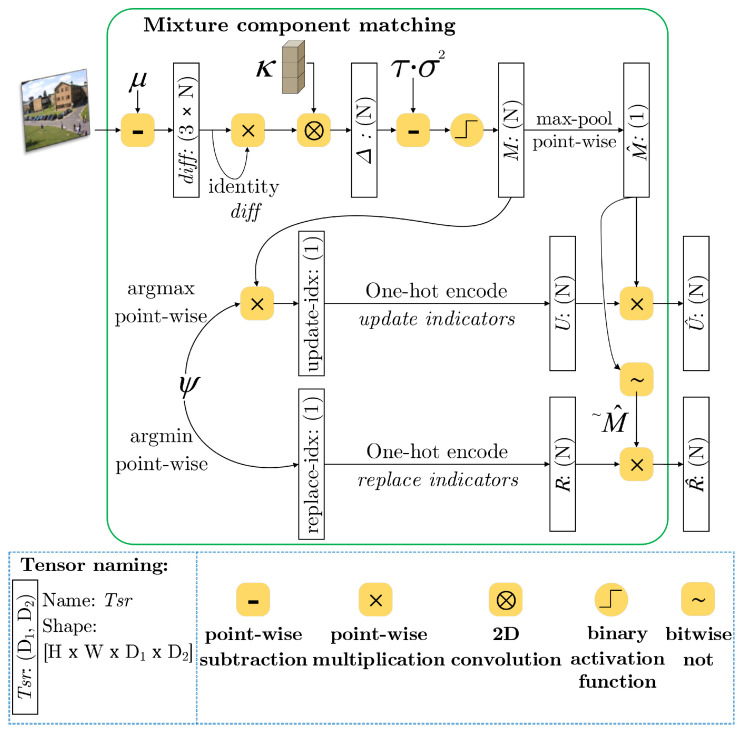
The tensor architecture of mixture component matching.

The bottleneck of iterative pixel-wise update in traditional approaches is the sequential pixel-by-pixel update with various conditions for both the enact and changeable components. In this work, we use tensors to present mixture parameters where the number of Gaussian components at each image point is settled as a finite factor. In model learning, the parameters are incrementally adjusted to best fit the temporal density of the observed scenes. However, depending on the incoming intensity values, we need to consider an inception of a new distribution or an act of superseding the most obsolete component in each mixture. To formulate this strategy in our tensor-driven architecture, we produce two other tensors of mixture component-indexed masks in parallel regarding two scenarios of the intensity-distance constraint at each image point x, as summarized in Figure 2.

- *If a match is found within N distributions:* we select the best-fit component with the highest value of ψ(x) via constructing a update-mask $\hat{U}$, which is presented by component-level multiplicative association of matching conventions in *M* and a high weight with a weak variance in terms of a depth-*N* one-hot map $\hat{U}\left(x\right)=\hat{M}\left(x\right)\cdot U\left(x\right)$, where, for $n\in \left[1,N\right]$:
  (5) $$U_{n}\left(x\right)=\left\{\begin{array}{c} 1,\;\mathrm{if}\;\underset{n^{\prime }}{argmax}\left[M_{n^{\prime }}\left(x\right)\cdot \psi_{n^{\prime }}\left(x\right)\right]=n\\ 0,\;\mathrm{otherwise} \end{array}\right.$$
- *If no match is found within N distributions:* we consider picking out either unmatched, non-existing distributions or those have least probable distribution in the mixture to initiate a new Gaussian. However, this manner is riddled with errors when selecting components having the least value of ωn,tωn,tσn,t2σn,t may falsely take up distributions that have been matched for EM updates. The replace-mask $\hat{R}$ can be fixed by employing the negation of $\hat{M}$ as $\hat{R}\left(x\right)=\left(\;\hat{M}\left(x\right)\right)\cdot R\left(x\right)$, where, for $n\in \left[1,N\right]$:
  (6) $$R_{n}\left(x\right)=\left\{\begin{array}{c} 1,\;\mathrm{if}\;\underset{n^{\prime }}{argmin}\left[\psi_{n^{\prime }}\left(x\right)\right]=n\\ 0,\;\mathrm{otherwise} \end{array}\right.$$

Next, statistical learning is directly performed on the model’s parameters via a computational flow of tensors. By making use of the $\hat{U}$ and $\hat{R}$ masks, we selectively alter the internal values of the μ, $\sigma^{2}$, ω and ψ tensors at each point. Following Equations (7)–(9), we first update the weight, variance, and mean to adapt the distributions that best match the prescribed criteria through over-relaxation. Simultaneously, we replace the least relevant distributions, or just plainly add new ones among pixel positions whose mixtures are without a match with the current input.

(7) $$\mu_{t+1}=\;{}^{∼}\hat{R}\cdot \left[\alpha \cdot \hat{U}\cdot I_{t}+\left(1-\alpha \right)\cdot \mu_{t}\right]+\hat{R}\cdot I_{t}$$

(8) $$\omega_{t+1}=\;{}^{∼}\hat{R}\cdot \left[\alpha \cdot \hat{U}+\left(1-\alpha \right)\cdot \omega_{t}\right]+\hat{R}\cdot \alpha$$

(9) $$\sigma_{t+1}^{2}=\;{}^{∼}\hat{R}\cdot \left[\sigma^{2}+\alpha \cdot \hat{U}\cdot \left(\Delta_{t}-3\sigma_{t}^{2}\right)\right]+\hat{R}\cdot {\bar{\sigma }}_{lower}^{2}$$

Equation (9) requires the differences between Δ and three times the variance. This is because of the fact that delta is the sum of vectorized square differences between the mean and input over three color channels RGB. The variance, given its single values, takes this into account and it is multiplied accordingly to properly become the common variance for all three color ranges. Following the update equations, it occurs that the more continuously a distribution gets selected for input data, the more its corresponding variance shrinks to fit. Consequently, frequently selected distributions become greatly restrictive in terms of matching for maintenance after a while. By setting a minimum values in the variance tensor, we are ensuring at least several degrees of freedom for its distributions, specifically at square units of intensity radius ${\bar{\sigma }}_{lower}^{2}$.

By definition, mixtures of Gaussians must satisfy the criteria:

(10) $$\sum_{n=1}^{N}P\left(G_{n,t}\right)=\sum_{n=1}^{N}\omega_{n,t}=1$$

Because the described update equations do not warrant such characteristics, we need to properly normalize the weights mixture-wise, whilst maintaining their relative orders, as denoted by Equation (11). Subsequently, Equation (12) renews the sorting-key tensor.

(11) $$\omega_{n,t+1}=\frac{\omega_{n,t+1}}{\sum_{n=1}^{N}\omega_{n,t+1}}$$

(12) $$\psi_{n,t+1}=\frac{\omega_{n,t+1}}{\sigma_{n,t+1}}$$

A background can be extracted via selecting the means whose corresponding distributions have the highest sorting-key values of their mixtures, following Equations (13) and (14). This ensures that high-weighted, low-spread distributions are used for modelling.

(13) $$BG_{t}=\max_{n}\left(\mu_{n,t}\cdot {\hat{BG}}_{n,t}\right),\;\mathrm{for}\;n\in \left[1,N\right]$$

where background mapping is defined at each pixel x as:

(14) $${\hat{BG}}_{n,t}\left(x\right)=\left\{\begin{array}{c} 1,\;\mathrm{if}\;\underset{n^{\prime }}{argmax}\left[\psi_{n^{\prime },t}\left(x\right)\right]=n\\ 0,\;\mathrm{otherwise} \end{array}\right.,\;\mathrm{for}\;n\in \left[1,N\right]$$

Accordingly, the corresponding foreground mask of each input frame is extracted the from Gaussian mixtures at all pixel locations. We performed a similar action when matching distributions are found for an input. Specifically, we applied the binary function $\phi \left(\cdot \right)$ on the subtraction by a threshold ξ from the squared Mahalanobis distance between the input frame and background distribution. This essentially creates a foreground mask holding the value 1 at locations where the Mahalanobis distance of input when compared to background is consistently within an allowed threshold, and 0 otherwise. This threshold is purposed to allow filtering of motion noises (e.g., flowing river, waving tree) inherent to a scene by allowing for deviations of several units of standard deviation around the means of background distributions. To this cause, the threshold is a constant proportional to the average entropy of a scene:

(15) $$FG_{t}=\phi \left[{\left(I_{t}-BG_{t}\right)}^{2}-{\hat{\sigma }}_{t}^{2}*\xi \right]$$

where

(16) $${\hat{\sigma }}_{t}^{2}=\max_{n}\left(\sigma_{n,t}^{2}\cdot {\hat{BG}}_{n,t}\right),\;\mathrm{for}\;n\in \left[1,N\right]$$

### 3.3. Adaptive Learning for Scene Variation Threshold

In this section, we propose an adaptive thresholding scheme in order to alleviate problems that arise from one of TensorMoG’s advantages, its strong adaptiveness. From the algorithmically statistical perspective, TensorMoG indiscriminately considers all of the sequential data as valuable contextual inputs towards background modelling. This carelessness means that occurrences of erroneous data due to anomalies (e.g., illumination shifts, camera jitterings, color flashes, etc.), and especially from ones that span over a period of time, are detrimental to TensorMoG maintenance processes.

The approach employs Shannon entropy, drawn from information theory [38], in order to numerically estimate probabilistic uncertainties for a mixture of Gaussians for evaluation. Every distribution in TensorMoG belongs to a mixture, they are weighted inclusively between one and zero, and combining all of these weights mixture-wise sums to one. Through the assumption that video sequences provide stochastic data influxes into TensorMoG’s processes, these distributions can be viewed as random variables, each with a stochastic score that is denoted by its weight:

(17) $$\rho_{t}=-\sum_{n}P\left(G_{n,t}\right)\cdot \log\left(P\left(G_{n,t}\right)\right)=-\sum_{n}\omega_{n,t}\cdot \log\left(\omega_{n,t}\right)$$

The greater the entropy, the more ambiguous and inconsistent the image point. In order to specify frames that present short-term dynamic changes, we estimate the average entropy values $E_{t}$ of each frame at time *t* with spatial consistency.

(18) $$E_{t}=\frac{1}{H\times W}\sum_{x}\rho_{t}\left(x\right)$$

The background decision relies heavily on the values of these weights, where it is obvious by following the described equations of TensorMoG that, under the effects of anomalies, distributions that represent a true background will numerically decrease their weights in consideration of faulty non-background ones. This occurrence, in turn, is corrupting towards modelling, as it renders the Mixtures of Gaussian unstable and sensitive to changes, even causing them to lose their true background distributions and waste time regaining foothold. Because every scene experiences motions that are part of their backgrounds (e.g., static streets, moving trees, flowing streams, etc.) they establish entropy ranges that are statistically very unique. Even for a scene, there can be major factors, such as changes in lighting, view, or the inclusion–exclusion of big objects that completely morph background decisions, adding to the already insurmountable varieties of possible ranges for background-stable entropy. Therefore, it is obvious that capturing problematic entropy ranges poses an issue that is only efficiently addressable via an adaptive, online learning scheme. Thus, in order to facilitate compatibility for any given sequences of contextual data, corresponding to our entropy estimates $E=\left\{E_{1},E_{2},\ldots ,E_{T}\right\}$, we set an entropy threshold set of $\bar{E}=\left\{{\bar{E}}_{1},{\bar{E}}_{2},\ldots ,{\bar{E}}_{T}\right\}$ that loosely minimizes the loss function of $L(\bar{E})$ in Equation (19).

(19) $$L\left(\bar{E}\right)=\sum_{t}{\left({\bar{E}}_{t}-E_{t}\right)}^{2}$$

where

(20) $$\begin{array}{cc} {\bar{E}}_{t} & =\left\{\begin{array}{c} {\bar{E}}_{t-1}+\alpha \frac{\partial }{\partial {\bar{E}}_{t-1}}L\left(\bar{E}\right),\;\;\mathrm{if}\;E_{t}>{\bar{E}}_{t-1}\\ \alpha {\bar{E}}_{t-1}+\left(1-\alpha \right)E_{t},\;\;\mathrm{otherwise}. \end{array}\right. \end{array}$$

Equation (20) describes the threshold value $\bar{E}$ at time *t* that can act as a baseline against which the entropy *E* at each time step *t* can be evaluated. In order to allow for online adaptiveness, $\bar{E}$ must always be able to converge towards commonly present entropy ranges. Thus, when an entropy estimate $E_{t}$ is above its corresponding threshold ${\bar{E}}_{t-1}$, our solution utilizes the gradual learning of gradient descent. This convergence is gradual with a view to capturing the rocketing speed of entropy (i.e., |${\bar{E}}_{t}-E_{t-1}$| over one time-step) when the scene enters an unstable sequence, allowing for either the discard of that sequence completely in the case of errors, or smooth transitioning for changing backgrounds. When an estimate $E_{t}$ decreases to below ${\bar{E}}_{t-1}$, this is considered to be synonymous to the model’s increasing assertions of its background decisions, as it is observed mathematically that selected distributions increase their weights and overtake others. Hence, ${\bar{E}}_{t}$ ought to be approximately equal to the estimate of entropy in those cases to definitively establish the baseline. This is achieved by the relaxation technique that effectively renders the previous estimates of threshold to be much less relevant than new entropy estimates. Finally, by allowing for a small percentage of deviation, the strong threshold at each time step becomes $\bar{E}\times \left(1+\epsilon \right)$ and, whenever entropy surpasses that magnitude, an anomalous scenario is imminent for the corresponding time step.

### 3.4. High Variation Removal and Dynamic Scene Adaptation

In this section, we propose a strategy that coordinates TensorMoG with an online thresholding scheme, which helps to avoid distortions from anomalous updates and facilitate coherent modelling amidst variations. TensorMoG is very adaptive, but, since it does not discriminate its inputs, it is prone to prolonged noise effects (e.g., sudden flashes, camera shaking) that render erroneous predictions, as mentioned. Using online thresholding, it is possible to detect when these effects occur by finding rises in entropy. However, real background changes, such as due to lighting, also breed uncertainties in the form of entropy increases when the background shifts itself both mathematically and visually, and it can be mistakenly confused as a manifestation of prolonged noises. From these insights, our strategy is to provide a blend between an Updating state, which maximizes the capability of TensorMoG on consistent sequences of inputs, and a Suspending state, whose goal is to employ the adaptiveness of TensorMoG to assert background decisions through either disposable or valuable variations.

Updating state is an explicit modelling state, which, at each time step *t*, directly alters TensorMoG’s parameters. During this state, TensorMoG maintains a thresholding scheme along estimations of entropy in order to assess these motion-dependent scores. The state assumes gradual, slow contextual changes within a sequence of images, where TensorMoG adapts most fluidly. An otherwise case occurs when the entropy estimates exceed the threshold for a duration of time $\hat{t}$, engendering robustness for deciding when the assumption fails. We only consider a high variation that consecutively takes place during one-second period. This declares the flag signal for the start of a highly varying frame sequence, then TensorMoG stops updates its background entirely, saves its background parameters, and moves on to suspending state without discontinuing entropy estimations and threshold updates.

Suspending state only produces the immediate background that was constructed in updating state before its maneuver to the this state, and does not immediately alter background predictions. Essentially, this state only performs implicit updates to TensorMoG’s tensor variables. This state assumes the presence of unstable variations within the sequence that is being processed. The state is maintained in a similar way as an updating state, via the estimation of entropy and thresholds. When entropy estimates are under or equal to corresponding threshold values for a duration of time $\hat{t}$, the assumption of high variation fails. This entails that TensorMoG has adapted well enough towards a background whose representing Gaussian distributions are frequently selected, either by successfully converging towards a new scene or having thrown away bad variations and recovering to an old one. The durational factor $\hat{t}$ offers robustness in making that decision through sustainment of the cause. Subsequently, TensorMoG returns to Updating state, resuming normal operations.

**Figure 3:**
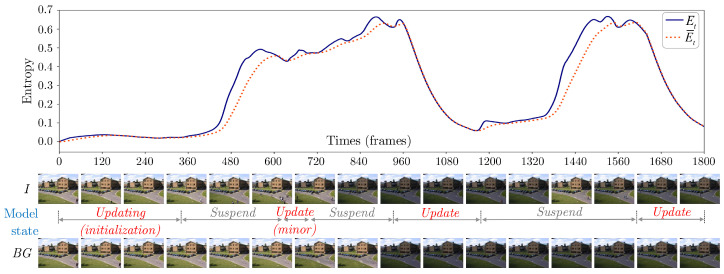
An illustration of adaptive threshold learning and dynamic scene adaptation with the image sequence *Dataset3Camera1* in the motion detection challenge *Illumination Changes* from the IEEE Scene Background Modelling Contest (SBMnet).

With this modelling strategy, foregrounds are extracted via Equations (15) and (16) throughout. However, for the suspending state, foregrounds are found by taking the Mahalanobis difference of an input image from the last background distribution that is predicted in an assumed stable sequence, specifically with $BG_{t}$ and ${\hat{\sigma }}_{t}^{2}$ constant and being carried over from the last prediction of the updating state. Addressing anomalous variations in such a way provides robustness towards making decisions, as every state switch requires durational sustainment of the cause. Algorithm 1 describes this approach, which extends TensorMoG into a tensor-driven model of both adaptiveness and selective variation removal. To illustrate, Figure 3 exemplifies adaptive threshold learning and dynamic scene adaptation of this work.
**Algorithm 1** Background modelling with frame-selective update1:Input2:$I_{t}$: the input frame.3:model: the TensorMoG model.4:Output5:model: frame-selective TensorMoG.6:**procedure**Process7: Begin8: state←Updating9: **for** each input frame $I_{t}$ **do**10:  Update model via Equations (7)–(9), (11) and (12)11:  Estimate $E_{t}$ via Equation (18)12:  Update ${\bar{E}}_{t}$ via Equation (20)13:  **if** state=Updating **then**14:   Update background image via Equation (13)15:   **if** $E_{t}\geq \left(1+\epsilon \right)\times {\bar{E}}_{t}$ **then**16:    **if** counter=fps **then**17:     Save background image18:     counter←0        ▹ for Suspending state19:     state←Suspending20:    **else**21:     counter←counter+122:    **end if**23:   **else**24:    counter←025:   **end if**26:  **end if**27:  **if** state=Suspending **then**28:   **if** $E_{t}\leq \left(1+\epsilon \right)\times {\bar{E}}_{t}$ **then**29:    **if** counter=fps **then**30:     counter←0         ▹ for Updating state31:     state←Updating32:    **else**33:     counter←counter+134:    **end if**35:   **else**36:    counter←037:   **end if**38:  **end if**39:  Compute foreground via Equation (15)40: **end for**41:**end procedure**

## 4. Results and Discussion

### 4.1. Experimental Setup and Dataset Selection

In this section, we demonstrate that our proposed TensorMoG is effective and can compete with existing state-of-the-art methods in terms of accuracy and processing time. TensorMoG was compared with state-of-the-art of methods that adopted an unsupervised learning approach: GMM—Stauffer & Grimson [19], GMM—Zivkovic [37], LOBSTER [39], SuBSENSE [40], SJN-Multiclue [41], IBMS [42], PAWCS [15], SWCD [43], PBAS [44], FTSG [26], and BMOG [23]. In addition, regarding the strategy of supervised learning, we make a comparison with some of recent deep-learning-based methods that are related to the problem of change detection and background modelling: DeepPBM [45]. DeepBS [11], Cascade CNN [12], STAM [34], FgSegNet [9], and FgSegNet_v2 [35].

In our experiment, *N* is empirically and heuristically determined by the TensorMoG’s capability of modeling constantly evolving contexts (e.g., moving body of water) under the effects of potentially corruptive noises, such that the framework utilizes reasonable space and time complexity, where *N* is a scaling factor. Because *N* is greater, there are many GMM components that are either unused or they simply capture the various noises presenting within the imaging sequences, especially contextual dynamics. This is because the background component would only revolve around the most frequently occuring color subspaces to draw predictions; so, the extra components would serve as either placeholders for abrupt changes in backgrounds, be empty, or capture intermittent noises of various degrees. In addition, using a greater value of *N* would increase the probability that the observed intensiy values match with the Gaussian mixture. Nevertheless, the extent to small non-matching probability mathematically depends on the minimum variance that is configured. In practice, noise Gaussian components in GMMs are pulse-like, as they would appear for short durations, and are low-weighted because they are not as often matched as background components. Hence, when *N* is great enough, the cumulative combination of non-background Gaussian components in the mixture would greatly reduce the non-matching probability if certain minimum variances are introduced. Otherwise, the pulse-like distributions would have negligible effects and the probability of finding no matches could still be high on assumption of completely random color vector input. In our work, we have emperically chosen N=3, so that TensorMOG can capture the high-frequency GMM components that are representative of backgrounds; it can also address certain degrees of noises with its non-background components whilst refraining from introducing too many computational overheads. Table 1 illustrates a set of TensorMoG’s parameters.

Retrieving relevant information from recently comprehensive account of public video datasets for background subtraction and foreground detection [46], our evaluations are conducted while using four publicly available benchmarking datasets:

– *CDnet 2014* [47]: the large-scale database of videos for motion detection across various scenarios.
– *CAMO-UOW* [48]: a dataset specifically designed for evaluating the segmentation of camouflaged motion changes where foreground objects are of similar color as that of the background. This is less frequently used for benchmarking algorithms.
– *SBMnet* [49]: the Scene Background modelling.NET dataset contains realistic videos to investigate modelling background scenes.
– *SBI* [50]: the Scene Background Initialization (SBI) dataset is a set of image sequences where background images are occluded by foreground objects, which are designed to evaluate the formulation of background under a variety of challenging conditions.

In measuring the quality of our method along with others in terms of changes and motions, we adopt the common evaluation metrics that require computations of a confusion matrix: Precision, Recall, F-Measure, Specificity, False-Negative Rate (FNR), False-Positive Rate (FPR), and Percentage of Wrong Classification (PWC) [51]. The overall results are similarly extracted from combining all sequence-based confusion matrices. Benchmarks using CDnet and CAMO-UOW employ these metrics for foreground predictions against provided ground-truths. Similarly for modelling background scenes, common and specific evaluation metrics are used, including the Average Gray-level Error (AGE), percentage of Error Pixels (pEPS), and percentage of Clustered Error Pixels (pCEPS), where, for all three, lower is better; and, the Multi-Scale Structural Similarity Index (MS-SSIM), Peak-Signal-to-Noise-Ratio (PSNR), and Color-image Quality Measure (CQM), where higher scores mean better. Among the metrics, because AGE, pEPS, and pCEPS are sensitive to even small variations and require exactness, they are practically less informative to demonstrate similarity measures against ground-truths than MS-SSIM. Evaluations with SBI and SBMnet datasets employ these metrics for comparing background modelling results against the true backgrounds. We used the available implementation of these metrics that were provided by SBI and SBMnet challenges [49], but evaluated for all background predictions against their ground-truths and not just the cleanest prediction. The final result for a sequence is the average of all calculations for all frames against the sequence’s provided ground-truths. Lastly, we analyze all of the methods in terms of processing speed with the image resolution of 320×240.

### 4.2. Results on CDnet 2014 Benchmark

**Figure 4:**
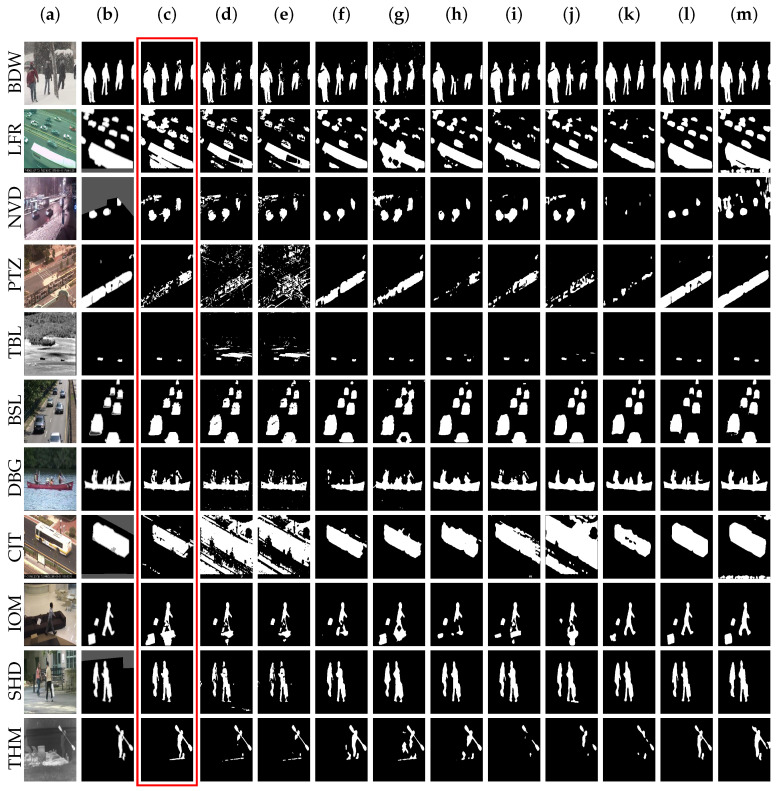
Visual quality comparison for foreground detection on some of video sequences in eleven categories from CDnet 2014. The columns include (**a**) input frame, (**b**) corresponding groundtruth foreground, (**c**) TensorMoG, (**d**) GMM—Stauffer & Grimson, (**e**) GMM—Zivkovic, (**f**) SuBSENSE, (**g**) SWCD, (**h**) PAWCS, (**i**) FTSG, (**j**) BMOG, (**k**) DeepBS, (**l**) FgSegNet_v2, (**m**) Cascade CNN.

With the large-scale CDnet2014 dataset, we demonstrate the effectiveness of our method by performing evaluations for all scenarios of differing multimodality and conditions of noises. Table 2 illustrates our F-measure comparison and Table 3 provides further details with regard to the recorded average of foreground metrics. Furthermore, Figure 4 provides a visual close-up of the compared methods’ motion extraction results. It can be seen that the proposed method takes the first place overall across the different scenarios and even several of the scenarios (i.e., *BDW*, *TBL*, *BSL*, *DBG*, *SHD*), whilst still in top-3 with *NVD*, *LFR*, and *THM* among the compared unsupervised methods. However, it is not without its weaknesses, as seen in the results of Table 3 for F-measure scores of *PTZ*, *NVD*, *CJT*, and *IOM*.

Firstly, TensorMoG is highly effective in foreground segmentation on common cases (the *BDW*, *BSL*, *TBL*, *DBG*, *SHD* and *THM* scenarios) whose dynamic contexts contain both constant ambient motions innate to the background, and object motions of interest. Holding top place in overall F-score with the scenarios (and top-3 on *THM*) regarding unsupervised approaches, TensorMoG demonstrates remarkable improvements over its GMM counterparts and, at times, even over the more computationally expensive approaches SuBSENSE, PAWCS, and SWCD by noticeable margins. For instance, whilst *BSL* sequences are effectively tackled by TensorMoG with clean backgrounds that are only slightly better than the SuBSENSE’s score at 0.9503, on the *DBG* scenario, our proposed approach is able to distinguish object motions from the background much better at 0.9325 than SuBSENSE at 0.8177. This suggests that the proposed model could capture locally varying degrees of dynamics in order to construct the overall backgrounds for selecting out raw irrelevant information predicted as foregrounds. TensorMoG’s accuracy is reduced when scene dynamics vary globally with spatial translations, like in *TBL*. This scenario also presents a great challenge for the compared approaches, because, while objects’ displacements can be captured, scene contextual variations may also be considered to be object motions due to the unpredictably translating background objects that are caused by turbulence from the camera perspectives. *THM* sequences present much better camouflaging effects than have been recently addressed by TensorMoG at 0.8380; these effects can be discussed along the next subsection with other experiments.

Apparently, TensorMoG is fairly erroneous at *LFR*, which is the scenario of great portions of motions for extraction due to its intermittent capturing of imaging signals. The similar situation is also seen at *CJT*, which perpetuates spatially locally translating imaging inputs due to the video recorder’s motions and, at *IOM*, where objects of interest exert intermittent motions throughout the observational views. TensorMoG’s motion predictions on the *LFR* scenario contain “holes” in the foreground map and they impose significant cumulative errors to the overall results, as shown in Figure 4. This is clarified by TensorMoG’s actual score of only 0.6852, whereas SWCD’s detailed analysis can produce the overall score of 0.7374. Because *CJT* presents similar situations to *TBL*, but more enhanced in translated motions of background objects, our overall score is only 0.6594, being weighed down by a significant number of False Positives, and it is toppled by SuBSENSE’s 0.8152 with its locally adaptive sensitivity. For *IOM*, TensorMoG’s reduced accuracy of 0.6493 is expected, as the method’s adaptiveness considers stopped objects as part of the background after they have stayed within the observational area for a very long amount of time. Being designed to tackle stopped objects, the Flux-Tensor method produces better results, at 0.7891 overall, in the F-measure for the same scenario.

However, the results of TensorMoG also suggest poor performance on *NVD* scenes, where night-lightings and moving objects intertwine on the motion maps, and its worst scores are on the *PTZ* scenario, with abrupt camera motions. Other methods, even some supervised ones, are not able to perform at more than 0.8 in F-measure with *NVD*. Like other unsupervised, TensorMoG is riddled with many False Negatives in this scenario, because night-lightings present very high degrees of motion noises. However, TensorMoG was able to stay in top-2 at 0.5604, and only slightly lower than SWCD’s 0.5807 among the unsupervised approaches. Among all scenarios, *PTZ* presents the greatest challenge for TensorMoG, as its F-score is only at 0.2626. *PTZ* also emerges as a challenge for all of the compared unsupervised methods, where the greatest score is only 0.4615 by PAWCS, along with the supervised DeepBS at only 0.3133. This is because the changes in camera perspectives can turn historically recorded information quickly obsolete. Thus, TensorMoG and methods utilizing durational information (e.g., DeepBS with background learning on pre-generated labels) are faulted by the lack of relevant information for correct predictions. Figure 4 also showcases this.

Nevertheless, the overall result on all scenarios of TensorMoG, which is top-1 among the unsupervised approaches and it is within top-3 for eight out of 11 CDnet2014 scenarios in the F-measure. In addition, we draw, from Table 3, more detailed evaluation of the proposed method in terms of Recall, Specificity, FPR, FNR, PWC, and Precision. Apparently, TensorMoG is the most precise among the compared unsupervised methods, at 0.8215, with an overall Recall score that is third best, at 0.7772. Furthermore, error rates, such as FPR at 0.0107, FNR at 0.2228, and PWC at 2.3315, do not deviate too far from the top scores at FPR-0.0051 of PAWCS, FNR-0.1876 of SuBSENSE, and PWC-1.1992 of PAWCS. All of which illustrate the proposed method’s general utility in practice without requiring intensive pretraining.

For supervised methods, we include, for comparative discussion in both Table 2 and Figure 4, the experimental results of methods utilizing DNNs. Specifically, we include a group of scene-specific DNNs that can detect motion changes by training with 200 frames per video sequence FgSegNet, FgSegNet_v2, and Cascade CNN, and another group consisting of multi-scene-single-model methods, DeepBS and STAM, which were trained with 5% of data from CDnet2014. From the results of Table 2 and Table 3, these DNN methods are capable of producing highly accurate results for motion segmentation across varying degrees of scenarios, aside from DeepBS, whose results are dependent on its background process, their accuracy results significantly surpass those of TensorMoG. They do this by learning the mapping function from series of imaging inputs to the expected motion maps, where their heavy architectural structures of parameters are found at extrema in the target-prediction function of difference. Thus, they are very capable of capturing useful, high-level motion patterns of their domain for extracting predictions. This is demonstrated best with scene-specific methods FgSegNet and FgSegNet_v2’s almost perfect overall results, at 0.9770 and 0.9847 in F-measure, and that of Cascade CNN at 0.9209, along with their respective self-explanatory results for other metrics (e.g., Specificity, FPR, FNR, PWC, etc.). Whereas, the scene-specific approach’s goal is to slightly overfit to the video of interest without the requirement of generalizing to any other contexts, thereby conceiving multiple models for multiple video scenarios of a dataset, the multi-scene-single-model approach adopted by DeepBS and STAM learns to generalize by sampling on the dataset for training. Specifically, DeepBS and STAM each only needed a single model for 53 sequences in CDnet2014 via training on 5% of the given data in order to capture patterns in the domain. DeepBS’s accuracy performance in terms of F-measure is weighed down by reduced scores in *LFR* at 0.6002, *NVD* at 0.5835, *IOM* at 0.6098, and *PTZ* at only 0.3133, thereby resulting in an overall lowest score of 0.7458 among the supervised approaches. DeepBS’s accuracy performance in terms of F-measure is weighed down by reduced scores in *LFR* at 0.6002, *NVD* at 0.5835, *IOM* at 0.6098, and *PTZ* at only 0.3133, thereby resulting in an overall lowest score of 0.7458 among supervised approaches. As this overall result is even lower than that of TensorMoG, it suggests that the DeepBS is still under-trained with only 5% of data, especially on *LFR* with a large number of intermittent objects, *NVD* with high degrees of lighting noises, *IOM* where there may be assimilation of stopped objects into DeepBS background model, and *PTZ* where the perspectives vary so much. In a similar way, on the *LFR* and *NVD* scenarios, STAM achieved F-measure scores of 0.6683 and 0.7102, respectively. Despite being less trained on *PTZ* than other scene-specific approaches, STAM’s high grading of 0.8648 in F-measure far surpasses those of unsupervised methods, which suggests the great mapping correctness reaped from heavy computations.

The numerical outcomes from other metrics (Recall, Specificity, etc.) further clarifies the advantage that DNN models hold. Aside from DeepBS, which relies on the performance of its background model, others demonstrated top input-output learned mapping capability at roughly 0.9 in Precision, Specificity, and Recall or above, and very low error rates. Nevertheless, like Cascade CNN and FgSegNets, which overfit to a video sequence and benefit from its results, the generalization that is obtained with the single-model approach is dataset-specific, as demonstrated in [34] with reduced accuracy when switching datasets. Therefore, in practice, if the contextual perspectives significantly deviate from what have been trained (i.e., switching perspectives, outdoor weather changed, camera repositions, etc.), these multi-scene-single-models would still require hardware-intensive, data-driven fine-tuning on the new views in order to incorporate their patterns into the architectural parameters. Consequently, the DNN technologies are still challenged by the trade-offs between accurate generalization and computational expenses, given the availability of data.

### 4.3. Results on CAMO-UOW Benchmark

In the same way, we analyze the performances of all methods with the Camouflaged Object dataset. Our goal for testing with this dataset is to demonstrate the effectiveness of the proposed method for extracting objects of interest under camouflaging effects, notable conditions that often hinder performance. We filtered out several sequences to only leave three sequences of grayscale and three of RGB color spaces. The reason for this is to provide meaningful discussion of the overall results, as not only the overall values will vary little when compared to those extracted with separate sequences, but it also allows for more thorough inspection of cases with the experimented methods. For this dataset, because SWCD was only tuned for CDnet via its own set of hyperparameters, we could not objectively provide its predictions for this dataset. On account of the fact that this dataset consists of imaging resolutions that vary from 1600×1200 to 1920×1080 pixels, the computational expenses incurred for retraining supervised methods on video sequences are very prohibitive. Consequently, objective measurements for DeepBS, FgSegnet, FgSegnet_v2, and STAM could not be provided by us on the dataset. In addition, we have to replace FTSG and BMOG with LOBSTER, PBAS, SJN-Multiclue, and IMBS, which are also unsupervised state-of-the-arts due to encountered objective implementational issues for this dataset. Because our experiments with camouflaging effects only serve to emphasize our proposed model’s generalization capability to the problematic case, the comparative evaluation with the selected methods can still provide objective analysis without any lack of rigour.

For this dataset, overall, our proposed method is third best in terms of F-measure, as illustrated in Table 4. TensorMoG is comparable to both LOBSTER (best) and SuBSENSE (second best) in these cases. Particularly, it can be seen that TensorMoG performs very well on *Video 03*, *Video 09*, and *Video 10* at around 0.8 and, especially, *Video 07* at 0.9131. These sequences are similar, as each has moving people that are partially dressed in similar color as the background. Although it is surpassed by the top methods (LOBSTER and SuBSENSE) and PAWCS in *Video 07*, *Video 09*, and *Video 10*, where there exists small but still humanly distinguishable color differences in the camouflaging areas, TensorMoG is still competitive against them, because it can better address the outdoor camouflaging effects, with the addition of lighting noises and a stopped object, in *Video 03* at 0.8090. Cases of marginal color deviations are also demonstrated among the methods in *Video 01* and *Video 05*, where people are completely dressed in indistinguishable colors from the backgrounds. All of the compared methods are still challenged in tackling these scenarios, as even the best result of *Video 05* from GMM—Stauffer & Grimson is only 0.6666. Like related methods, TensorMoG is also challenged, where its results are 0.7311 on *Video 01*, despite being third-best, and only 0.6066 on *Video 05*. This suggests a weakness of the proposed method in the presence of high degrees of overlaps. Nevertheless, the overall score of 0.7944 still suggests TensorMoG’s reasonable utility in practical contexts.

An explanation for TensorMoG performance is that since mixtures’ top distributions are trained to initialize the background, their variances become smaller as their weights approach one, thereby making TensorMoG more sensitive to variations. However, difficult cases that are common to *Video 05* are like what happened at the 160th frame of *Video 01*, where all of the methods struggle to figure out foreground components that are indistinguishable from the canvas behind. This suggests a weakness of the proposed method. Because all of the variances of TensorMoG’s mixtures have a lower-bound in order to always allow certain degrees of color variations, then, if an object’s color compared to background is too similar, it will be assimilated as a background value.

**Figure 5:**
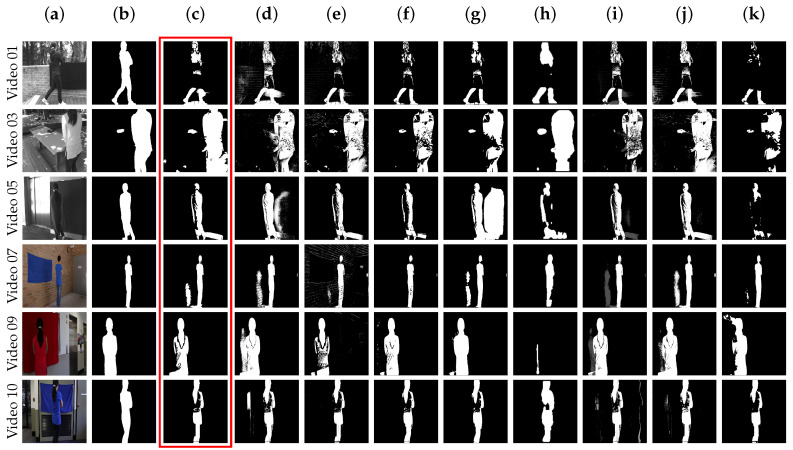
Visual quality comparison on six video sequences of camouflaged moving foreground detection in CAMO-UOW dataset. The columns include (**a**) input frame, (**b**) corresponding groundtruth foreground, (**c**) TensorMoG, (**d**) GMM—Stauffer & Grimson, (**e**) GMM—Zivkovic, (**f**) LOBSTER, (**g**) SuBSENSE, (**h**) SJN-Multiclue, (**i**) IMBS, (**j**) PBAS, (**k**) PAWCS.

At closer inspection, apparently, TensorMoG’s prediction results of colored images average higher than those of grayscale images. This is objectively attributed to the fact that distance measures between input and mixture distributions are in Euclidean space, where intensities and information completeness of colored images are better emphasized. Regardless of color spaces, it can be said that TensorMoG’s handling of foreground generation illustrates good improvements over other GMM models in many cases. Firstly, as demonstrated with *Video 05* and *Video 09* from Figure 5, it provides considerable removal of shadow effects whilst maintaining a high degree of granularity in terms of color changes. This is unlike the GMM version of Stauffer & Grimson, where, despite its capability of distinguishing granular color variations with the same video, object shadows are also captured from the sensitivity. Secondly, another advantage of TensorMoG is the provision of clean foregrounds, where not only foreground predictions are well-connected, but small noises are also removed (as compared to the GMM of Zivkovic), as shown in *Video 03* of Figure 5 with sunlight variations through the leaves, and objectively drawn from the corresponding F-measure. Thirdly, also with *Video 03*, due to TensorMoG’s mechanism of selective updates, it was able to capture the placed object on the table by resorting to the last stable background as entropy increases, unlike how the GMM of Stauffer & Grimson considers the plate part of the background so quickly.

The overall results are shown in Table 5 for elaborations with all other metrics. The proposed method at its worst is in fifth place against the more computationally expensive SWCD, PAWCS, LOBSTER, and SuBSENSE. Upon close inspection, it can be seen that TensorMoG’s overall results on performed metrics is very balanced, and they do not deviate too far from those at the top with tolerable error magnitudes. On the other hand, TensorMOG has slightly lower recall and higher FNR when compared to those of GMM—Stauffer & Grimson, which may be attributed to the fixed lower-bound mentioned that leads to missing minuscule differences between an object and its background. Nevertheless, as a GMM-based architecture for background modeling, TensorMoG scores illustrate good improvements over both its GMM counterparts at precision, specificity, FPR, and PWC, which suggests cleaner foregrounds with filtered noises and light shadows. It can be concluded that, although TensorMoG may not be the best at addressing camouflaging effects, its utility is evidently still within the practical domain.

### 4.4. Results on SBI and SBMnet Benchmark

The scene background modelling datasets SBI and SBMnet are used, as we compare the performances of different algorithms while using the discussed metrics for background construction. By default, there are several methods that inherently do not model background scenes (SJN-Multiclue, PBAS and SWCD) and thus are left out. We experimented on all of 13 video sequences in SBI and 10 video sequences in SBMnet with labelled grouthtruth of background images. The results on SBI and SBMnet datasets are summarized in Table 6 and Table 7, respectively.

The generation of backgrounds using TensorMoG is generally in the range from second to fourth best with its simple calculations. The scores of all GMM-based models (TensorMoG and GMM counterparts) are competitive with one another. However, they fall slightly short of IMBS and, at times, SuBSENSE with these durationally short sequences, due to initialization properties. Specifically, if the first frame, which biasedly provides the first mixture distributions, greatly deviates from the true background, then the GMM-based models require enough time to recover, otherwise the predictions will be riddled with faults. However, the GMM model that was devised by Zivkovic and, especially, that of Stauffer & Grimson, can still be considered to be the top performing approaches. This is based on the fact that, in terms of MS-SSIM (the structural similarity measure to evaluate perceived visual distortions), PSNR (the ratio between the maximum possible power of a signal and the power of corrupting noise), and CQM (the metric based on a reversible transformation of the YUV color space and on the PSNR computed in the single YUV bands), which are the especially designed imaging quality metrics for background evaluation, they are hardly surpassed with their mathematical rigour on pixel-wise evaluations.

Unlike SuBSENSE, whose performance evaluation scores greatly differ between the two datasets, it can be seen that TensorMoG is capable of maintaining balanced measurements for all metrics, such as Average Gray Error at 13.5404 for SBI versus that at 13.8699 for SBMnet across the separate corresponding sequences. With AGE, pEPs, and pCEPS measuring, the strictly precise similarity between background prediction and groundtruth in the grayscale color space, TensorMoG’s performance errors at constructing backgrounds for image understanding at around second–fourth place are tolerable, given that the input resolutions for SBI vary from 144 × 144 to 800 × 600 pixels, and those of SBMnet vary from 200 × 164 to 720 × 576 pixels. On the other hand, whilst IMBS produces the lowest errors in terms of these precise metrics, its perceived background predictions are still afflicted with motion features. This is best indicated by measurements that are made with the structural similarity metric MS-SSIM.

**Figure 6:**
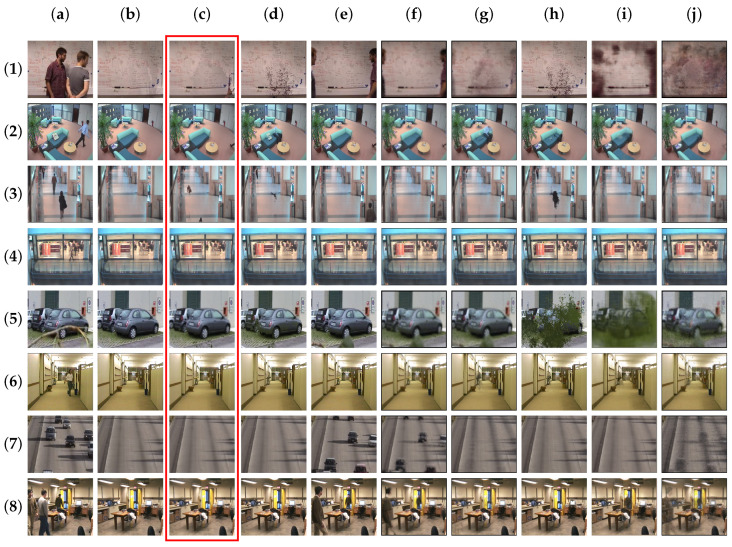
Visual quality comparison for background modelling on video sequences in SBI dataset. Some of SBI sequences: (**1**) sequence *Board*, (**2**) sequence *Candela_m1.10*, (**3**) sequence *CAVIAR1*, (**4**) sequence *CAVIAR2*, (**5**) sequence *Foliage*, (**6**) sequence *HallAndMonitor*, (**7**) sequence *HighwayI*, (**8**) sequence *HumanBody2*. The columns include (**a**) input frame, (**b**) groundtruth, (**c**) TensorMoG, (**d**) GMM—Stauffer & Grimson, (**e**) GMM—Zivkovic, (**f**) LOBSTER, (**g**) SuBSENSE, (**h**) IMBS, (**i**) DeepPBM, and (**j**) PAWCS.

**Figure 7:**
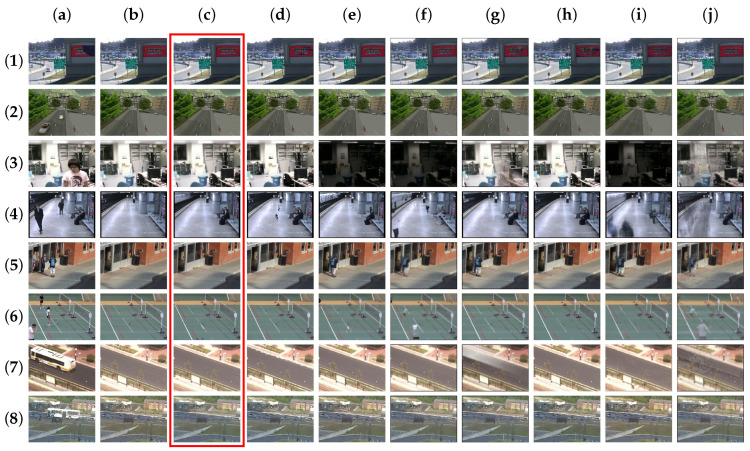
Visual quality comparison for background modelling on video sequences in SBM dataset. Some of SBM sequences: (**1**) background motion: sequence *advertisementBoard*, (**2**) basic: sequence *511*, (**3**) illumination changes: sequence *CameraParameter*, (**4**) intermittent motion: sequence *AVSS2007*, (**5**) intermittent motion: sequence *busStation*, (**6**) jitter: sequence *badminton*, (**7**) jitter: sequence *boulevard*, (**8**) very long: sequence *BusStopMorning*. The columns include (**a**) input frame, (**b**) groundtruth, (**c**) TensorMoG, (**d**) GMM—Stauffer & Grimson, (**e**) GMM—Zivkovic, (**f**) LOBSTER, (**g**) SuBSENSE, (**h**) IMBS, (**i**) DeepPBM, and (**j**) PAWCS.

DeepPBM, which engages a variational autoencoder (VAE) framework of DNNs in learning compressed representations of visual features in video sequence, is taken into consideration in this matter. We perform a training this architect from scratch with a batch size of 200, the dimension of the latent variables d=30. The background outputs that are constructed by DeepPBM are approximately satisfied with respect to the labelled groundtruth regarding video sequences with few variations like the sequence *Candela_m1.10*, *CAVIAR1* in SBI and the sequences *511*, *BusStopMorning*. However, the adaptation of this unsupervised probabilistic model estimation with VAE is not really sufficient in situations with drastic scene dynamics and high variation in a short period of time within short sequence, like the sequences *Board*, *Foliage*, *HumanBody2* in SBI and the sequences *CameraParameter*, *badminton*, and *boulevard* in SBMnet. The absence of a large-scale video sequence makes statistical regularities in this model weak to acquire the true background of scenes. When considering the MS-SSIM metric for both the SBI and SBMnet dataset, DeepPBM achieves a groundtruth similarity of 77.66% and 78.00%, respectively. Meanwhile, with an online statistical learning, our proposed TensorMoG takes the first and second place in MS-SSIM measurement, respectively, for these two dataset. Moreover, we maintain the third-lowest signal-noise ratio PSNR for both of the datasets, which is analyzed with provided background groundtruth. Figure 6 and Figure 7, respectively, illustrate the visual comparison for background modelling on the datasets SBI and SBMnet.

### 4.5. Speed Evaluation

Our proposed framework, along with DNN approaches, was implemented with CUDA on an NVIDIA GTX 1070 Ti or similar. The experimented unsupervised methods for comparison were based on the configuration of an Intel Core i7 with 16 GB RAM. We compare all of the methods’ processing time and summarize the results presented in Table 8. It can be seen that, in addition to being reliable, TensorMoG is also extremely fast. It is second to only the GMM of Zivkovic among all of the tested algorithms in terms of speed. However, as seen previously, this variant of background modeling is overshadowed by our model in the task of foreground extraction, due to its sensitivity to various noises. On the other hand, FgSegNets, Cascade CNN, and STAM outperform TensorMoG in terms of accuracy are now overthrown by the extent to which TensorMoG is faster. Because they are more focused towards providing accurate results, they are very lacking in processing speed, due to the great number of architectural parameters. In comparison, although TensorMoG is a worse than them in accuracy, our model is at least 10 times faster. Essentially, TensorMoG is the most balanced approach among all of the experimented methods in addressing the dilemma between accuracy and processing speed. Our proposed method is capable of maintaining good accuracy scores throughout the experimented datasets, whilst performing at highly superior processing speed than other approaches of top accuracy ranking. Furthermore, due to the nature of its parallel processing, TensorMoG is also able to process signals of multiple cameras at the same time via tensor concatenation. With this feature, along with its accuracy and impressive speed, TensorMoG is capable of providing a major advantage for industrial traffic surveillance systems.

### 4.6. Experimental Summary

Our experimental results indicated that the framework is robust against various situations for producing satisfactory foreground segments and background views. Its adaptiveness from the light-weighted architecture evidently suggests the capability for generalizations across scenes in the unsupervised manner without requiring prior data for pretraining. Specifically, whilst the proposed model presents marked improvements over the GMM-counterparts BMOG, GMM–Stauffer & Grimson, GMM–Zivkovic across the diverse array of scenarios (i.e., dynamic backgrounds, weather conditions, low framerate, etc.), it is also capable of surpassing the more sophisticated approaches SuBSENSE, SWCD, PAWCS, and FTSG, overall, on the CDnet2014 dataset. TensorMoG has also illustrated its utility in addressing the camouflaging effects for motion analysis with CAMO-UOW, being, overall, third-best and slightly numerically worse than LOBSTER and SuBSENSE. Furthermore, in terms of background construction, the results obtained by TensorMoG are only structurally second to GMM–Stauffer & Grimson or even the best among compared methods, as indicated by the MS-SSIM metric. Coupled with its very impressive speed, TensorMoG can be practically deployed across multiple outdoor and indoor real-time video scenes. Regarding this technological aspect, whereas the unsupervised models when compared with TensorMoG in CDnet2014, CAMO-UOW, SBI, and SBM follow the sequentially iterative paradigm of computations (even Flux-Tensor, despite its tensor-driven framework), TensorMoG is designed in order to utilize modern parallel computing technologies. On the speed test, TensorMoG is at 302.5261 frames-per-second, and is only surpassed by the performance of the less accurate GMM–Zivkovic. Nevertheless, aside from the great balance between accuracy and speed across scenes, the method is also shown to be challenged under certain ruinous effects (e.g., camera jitters in *CJT*, motion noises in *NVD*, changing perspectives in *PTZ* of CDnet2014, etc.) that would require further study.

In comparison with supervised approaches, it has been showcased that TensorMoG’s unsupervised statistical learning cannot outperform the data-driven, learned mapping of Cascade CNN, STAM, FgSegNet, and FgSegNet_v2, which are capable of finding detailed, high-leveled patterns across multiple scenarios, in terms of accuracy. Because DeepBS may be hindered by corrupted background models in certain scenarios, its performance being outperformed cannot be objectively viewed as an advantage of TensorMoG. Whereas, Cascade CNN and FgSegNets overfit to a video sequence and benefit from its results, the generalization that is obtained with the single-model approach with DeepBS and STAM is dataset-specific, which suggest further intensive training with more labeled data if contextual perspectives are significantly changed in practice (e.g., camera reposition) in order to incorporate new information. Thus, TensorMoG’s actual advantages over the supervised methods are its compromise in accuracy for low computational burdens, not requiring hardware-intensive pre-training on manually sampled data whilst being highly generalizable. This advantage can be emphasized in real-time, constantly evolving scenes, where deploying the light-weight TensorMoG would provide an edge in factoring out motions adaptively.

## 5. Conclusions

In this work, we proposed a novel framework of a tensor-driven GMM for background modelling and extended it with a mechanism of selective scene adaptation. Our most significant contribution in this study is to address the problem of pixel-wise methods by parallelizing statistical learning in spatial image resolution, whilst maintaining statistical adaptation. We also evaluate dynamic variations by estimating the entropy of each scene with an online, adaptive threshold learning approach.

Our strategy presents marked improvements over related studies in maximizing the stability of the background model while requiring low computational resources, since mixture learning only performs with background-stable situations. Our experimental results indicated that the framework came with greatly balanced compromise between background modelling and change detection by producing satisfactory foreground segments throughout multiple outdoor and indoor video sequences, along with very impressive speed.

On the other hand, it is clear that TensorMoG does not perform well in scenarios where there is a lack of information for background construction, high degrees of motion noises, or there are spatial translations in imaging signals. Our future work seeks to incorporate convolutional supervised training for spatio-temporally local smoothing and filtering in the tensor-driven framework, as this is obviously due to the pixel-wise statistical learning approach with fixed learning rate.

## Figures and Tables

**Table 1:** Parameters in the TensorMoG.

*N*	Number of Gaussian components	3
α	Learning rate	0.015
ϵ	Margin of entropy error	0.025
τ	Mixture component mapping threshold	36.0
ξ	Foreground matching threshold	25.0
${\bar{\sigma }}_{lower}^{2}$	Lower bound of distribution variance	48.0
${\bar{E}}_{t}$	Entropy threshold at time *t*	adapt during processing

**Table 2:** F—measure comparisons over the 11 categories in the CDnet 2014 dataset.

	Method	*BDW*	*LFR*	*NVD*	*PTZ*	*TBL*	*BSL*	*DBG*	*CJT*	*IOM*	*SHD*	*THM*	Overall
Unsupervised	**TensorMoG**	$0.9298_{\left(1\right)}$	$0.6852_{\left(2\right)}$	$0.5604_{\left(2\right)}$	0.2626	$0.7993_{\left(1\right)}$	$0.9738_{\left(1\right)}$	$0.9325_{\left(1\right)}$	0.6594	0.6493	$0.9488_{\left(1\right)}$	$0.8380_{\left(3\right)}$	$0.8226_{\left(1\right)}$
	GMM—S & G	0.7380	0.5373	0.4097	0.1522	0.4663	0.8245	0.633	0.5969	0.5207	0.7156	0.6621	0.5707
	GMM—Zivkovic	0.7406	0.5065	0.3960	0.1046	0.4169	0.8382	0.6328	0.567	0.5325	0.7232	0.6548	0.5566
	SuBSENSE	$0.8619_{\left(2\right)}$	0.6445	$0.5599_{\left(3\right)}$	$0.3476_{\left(3\right)}$	$0.7792_{\left(2\right)}$	$0.9503_{\left(2\right)}$	0.8177	$0.8152_{\left(1\right)}$	0.6569	$0.8646_{\left(3\right)}$	0.8171	$0.7408_{\left(3\right)}$
	SWCD	$0.8233_{\left(3\right)}$	$0.7374_{\left(1\right)}$	$0.5807_{\left(1\right)}$	$0.4545_{\left(2\right)}$	$0.7735_{\left(3\right)}$	0.9214	0.8645	0.7411	$0.7092_{\left(3\right)}$	0.8302	$0.8581_{\left(1\right)}$	$0.7583_{\left(2\right)}$
	PAWCS	0.8152	$0.6588_{\left(3\right)}$	0.4152	$0.4615_{\left(1\right)}$	0.6450	$0.9397_{\left(3\right)}$	$0.8938_{\left(2\right)}$	$0.8137_{\left(2\right)}$	$0.7764_{\left(2\right)}$	$0.8710_{\left(2\right)}$	$0.8324_{\left(2\right)}$	0.7403
	FTSG	0.8228	0.6259	0.5130	0.3241	0.7127	0.9330	$0.8792_{\left(3\right)}$	$0.7513_{\left(3\right)}$	$0.7891_{\left(1\right)}$	0.8535	0.7768	0.7283
	BMOG	0.7836	0.6102	0.4982	0.2350	0.6932	0.8301	0.7928	0.7493	0.5291	0.8396	0.6348	0.6543
Supervised	DeepBS	0.8301	0.6002	0.5835	0.3133	0.8455	0.9580	0.8761	0.899	0.6098	0.9092	0.7583	0.7458
	Cascade CNN	0.9431	0.8370	0.8965	0.9168	0.9108	0.9786	0.9658	0.9758	0.8505	0.9414	0.8958	0.9209
	STAM	0.9703	0.6683	0.7102	0.8648	0.9328	0.9885	0.9483	0.8989	0.9155	0.9663	0.9907	0.9651
	FgSegNet	0.9845	0.8786	0.9655	0.9843	0.9648	0.9973	0.9958	0.9954	0.9951	0.9944	0.9921	0.9770
	FgSegNet_v2	0.9904	0.9336	0.9739	0.9862	0.9727	0.9978	0.9951	0.9971	0.9961	0.9952	0.9938	0.9847

In each column, $Red_{\left(1\right)}$ is for the best, $Green_{\left(2\right)}$ is for the second best, and $Blue_{\left(3\right)}$ is for the third best. Experimented scenarios include bad weather (*BDW*), low frame rate (*LFR*), night videos (*NVD*), pan-tilt-zoom (*PTZ*), turbulence (*TBL*), baseline (*BSL*), dynamic background (*DBG*), camera jitter (*CJT*), intermittent object motion (*IOM*), shadow (*SHD*), and thermal (*THM*).

**Table 3:** Result of quantitative evaluation on CDnet 2014 dataset.

	Method	Average	Average	Average	Average	Average	Average
		Recall	Specificity	FPR	FNR	PWC	Precision
Unsupervised	**TensorMoG**	0.7772	0.9893	0.0107	0.2228	2.3315	0.8215
	GMM—Staffer & Grimson	0.6846	0.9750	0.0250	0.3154	3.7667	0.6025
	GMM—Zivkovic	0.6604	0.9725	0.0275	0.3396	3.9953	0.5973
	SuBSENSE	0.8124	0.9904	0.0096	0.1876	1.6780	0.7509
	SWCD	0.7839	0.9930	0.0070	0.2161	1.3414	0.7527
	PAWCS	0.7718	0.9949	0.0051	0.2282	1.1992	0.7857
	FTSG	0.7657	0.9922	0.0078	0.2343	1.3763	0.7696
	BMOG	0.7265	0.9813	0.0187	0.2735	2.9757	0.6981
Supervised	DeepBS	0.7545	0.9905	0.0095	0.2455	1.9920	0.8332
	Cascade CNN	0.9506	0.9968	0.0032	0.0494	0.4052	0.8997
	STAM	0.9458	0.9995	0.0005	0.0542	0.2293	0.9851
	FgSegNet	0.9836	0.9998	0.0002	0.0164	0.0559	0.9758
	FgSegNet_v2	0.9891	0.9998	0.0002	0.0109	0.0402	0.9823

**Table 4:** F—measure comparisons the CAMO-UOW dataset.

Method	*Video 01*	*Video 03*	*Video 05*	*Video 07*	*Video 09*	*Video 10*	Overall
**TensorMoG**	$0.7311_{\left(3\right)}$	$0.8090_{\left(2\right)}$	0.6066	0.9131	0.8252	0.8248	$0.7944_{\left(3\right)}$
GMM—Stauffer & Grimson	0.6334	0.6848	$0.6666_{\left(1\right)}$	0.8021	0.7760	0.7396	0.7169
GMM—Zivkovic	0.6298	0.7589	0.5818	0.7361	0.7558	0.8338	0.7345
LOBSTER	$0.7436_{\left(2\right)}$	0.7489	$0.6643_{\left(2\right)}$	$0.9312_{\left(1\right)}$	$0.8808_{\left(2\right)}$	$0.8908_{\left(2\right)}$	$0.8141_{\left(1\right)}$
SuBSENSE	$0.7761_{\left(1\right)}$	$0.8003_{\left(3\right)}$	0.6146	$0.9198_{\left(3\right)}$	$0.9139_{\left(1\right)}$	$0.9052_{\left(1\right)}$	$0.8130_{\left(2\right)}$
SJN-Multiclue	0.7163	$0.8101_{\left(1\right)}$	0.5773	0.7453	0.4560	0.8016	0.7282
IMBS	0.4212	0.4413	0.5233	0.4064	0.8142	0.7366	0.5458
PBAS	0.6941	0.7808	$0.6350_{\left(3\right)}$	0.8735	0.8238	0.7719	0.7700
PAWCS	0.6532	0.6274	0.5846	$0.9257_{\left(2\right)}$	$0.8379_{\left(3\right)}$	$0.8803_{\left(3\right)}$	0.7578

In each column, $Red_{\left(1\right)}$ is for the best, $Green_{\left(2\right)}$ is for the second best, and $Blue_{\left(3\right)}$ is for the third best.

**Table 5:** Result of quantitative evaluation on CAMO-UOW dataset.

Method	Average	Average	Average	Average	Average	Average
	Recall	Specificity	FPR	FNR	PWC	Precision
GMM—Stauffer & Grimson	0.8425	0.9714	0.0286	0.1575	3.5426	0.6239
GMM—Zivkovic	0.7516	0.9834	0.0166	0.2484	2.8930	0.7182
LOBSTER	0.7242	0.9969	0.0031	0.2758	1.7613	0.9293
SuBSENSE	0.8285	0.9882	0.0118	0.1715	2.0290	0.7981
SJN-Multiclue	0.7420	0.9834	0.0166	0.2580	2.9494	0.7148
IMBS	0.4949	0.9824	0.0176	0.5051	4.3127	0.6085
PBAS	0.8558	0.9794	0.0206	0.1442	2.7213	0.6999
PAWCS	0.6471	0.9966	0.0034	0.3529	2.2021	0.9143
**TensorMoG**	0.8079	0.9873	0.0127	0.1921	2.2268	0.7813

**Table 6:** Comparison on the Scene Background Initialization (SBI) dataset.

Method	Average	Average	Average	Average	Average	CQM
	AGE	pEPs	pCEPS	MS-SSIM	PSNR	
GMM—Stauffer & Grimson	15.7189	$0.1275_{\left(2\right)}$	0.1018	$0.8639_{\left(2\right)}$	$26.1304_{\left(1\right)}$	$26.9061_{\left(1\right)}$
GMM—Zivkovic	$12.9308_{\left(2\right)}$	$0.1342_{\left(3\right)}$	$0.0995_{\left(3\right)}$	0.8554	$23.5434_{\left(2\right)}$	$24.3670_{\left(2\right)}$
LOBSTER	19.2193	0.2166	0.1069	0.8457	17.5133	18.6786
SuBSENSE	19.5009	0.2367	0.1353	$0.8562_{\left(3\right)}$	18.0343	19.2097
IMBS	$6.2631_{\left(1\right)}$	$0.0503_{\left(1\right)}$	$0.0299_{\left(1\right)}$	0.4821	14.2453	14.6877
DeepPBM (d=30)	18.9370	0.2158	0.1453	0.7766	21.6669	22.5271
PAWCS	25.0482	0.3244	0.1752	0.7800	17.1190	18.2663
**TensorMoG**	$13.5404_{\left(3\right)}$	0.1566	$0.0912_{\left(2\right)}$	$0.8746_{\left(1\right)}$	$23.1693_{\left(3\right)}$	$24.0963_{\left(3\right)}$

In each column, $Red_{\left(1\right)}$ is for the best, $Green_{\left(2\right)}$ is for the second best, and $Blue_{\left(3\right)}$ is for the third best.

**Table 7:** Comparison on the Scene Background Modelling (SBMnet) dataset.

Method	Average	Average	Average	Average	Average	CQM
	AGE	pEPs	pCEPS	MS-SSIM	PSNR	
GMM—Stauffer & Grimson	$10.1010_{\left(3\right)}$	$0.0890_{\left(3\right)}$	$0.0490_{\left(3\right)}$	$0.9001_{\left(1\right)}$	$24.9205_{\left(1\right)}$	$25.9607_{\left(1\right)}$
GMM—Zivkovic	15.3910	0.1435	0.0919	0.8561	$22.3682_{\left(2\right)}$	$23.4559_{\left(2\right)}$
LOBSTER	17.9441	0.1768	0.0904	$0.8640_{\left(3\right)}$	18.6872	19.9690
SuBSENSE	$6.3606_{\left(2\right)}$	$0.0737_{\left(2\right)}$	$0.0290_{\left(2\right)}$	0.4435	9.9956	10.6403
IMBS	$6.1321_{\left(1\right)}$	$0.0496_{\left(1\right)}$	$0.0189_{\left(1\right)}$	0.6317	17.3638	18.0909
DeepPBM (d=30)	18.4458	0.1918	0.1181	0.7553	21.0928	22.0530
PAWCS	18.6100	0.2461	0.1142	0.8076	18.8029	20.0561
**TensorMoG**	13.8699	0.1446	0.0745	$0.8769_{\left(2\right)}$	$22.1011_{\left(3\right)}$	$23.2473_{\left(3\right)}$

In each column, $Red_{\left(1\right)}$ is for the best, $Green_{\left(2\right)}$ is for the second best, and $Blue_{\left(3\right)}$ is for the third best.

**Table 8:** Processing speed evaluation among methods regarding resolution 320×240 (unit: fps—frames per second, higher is better).

GMM—Zivkovic	**TensorMoG**	IMBS	GMM—S & G	BMOG	PBAS
$419.9497_{\left(1\right)}$	$302.5261_{\left(2\right)}$	$197.7705_{\left(3\right)}$	119.6968	102	72.2722
DeepPBM (*d* = 30)	SJN-Multiclue	LOBSTER	FgSegNet	SWCD	FgSegNet_v2
64.0156	54.1471	26.0961	23	20.0608	18
SuBSENSE	Cascade CNN	PAWCS	STAM	FTSG	DeepBS
15.7168	12.5	12.1585	10.8	10	10

$Red_{\left(1\right)}$ is for the best, $Green_{\left(2\right)}$ is for the second best, and $Blue_{\left(3\right)}$ is for the third best.
